# Estimation of Gene Expression at Isoform Level from mRNA-Seq Data by Bayesian Hierarchical Modeling

**DOI:** 10.3389/fgene.2012.00239

**Published:** 2012-11-27

**Authors:** M. Bhattacharjee, Ravi Gupta, R. V. Davuluri

**Affiliations:** ^1^Department of Statistics, University of PunePune, India; ^2^Department of Mathematics and Statistics, University of HyderabadHyderabad, India; ^3^Center for Systems and Computational Biology, The Wistar InstitutePhiladelphia, PA, USA

**Keywords:** mRNA-Seq, isoform expression, Bayesian latent variable modeling, multi-sample comparison, Bayesian *t*-test, spike-n-slab method

## Abstract

mRNA-Seq is a precise and highly reproducible technique for measurement of transcripts levels and yields sequence information of a transcriptome at a single nucleotide base-level thus enabling us to determine splice junctions and alternative splicing events with high confidence. Often analysis of mRNA-Seq data does not attempt to quantify the expressions at isoform level. In this paper our objective would be use the mRNA-Seq data to infer expression at isoform level, where splicing patterns of a gene is assumed to be known. A Bayesian latent variable based modeling framework is proposed here, where the parameterization enables us to infer at various levels. For example, expression variability of an isoform across different conditions; the model parameterization also allows us to carry out two-sample comparisons, e.g., using a Bayesian *t*-test, in addition simple presence or absence of an isoform can also be estimated by the use of the latent variables present in the model. In this paper we would carry out inference on isoform expression under different normalization techniques, since it has been recently shown that one of the most prominent sources of variation in differential call using mRNA-Seq data is the normalization method used. The statistical framework is developed for multiple isoforms and easily extends to reads mapping to multiple genes. This could be achieved by slight conceptual modifications in definitions of what we consider as a gene and what as an exon. Additionally proposed framework can be extended by appropriate modeling of the design matrix to infer about yet unknown novel transcripts. However such attempts should be made judiciously since the input date used in the proposed model does not use reads from splice junctions.

## Introduction

### Background

**Figure 1:**
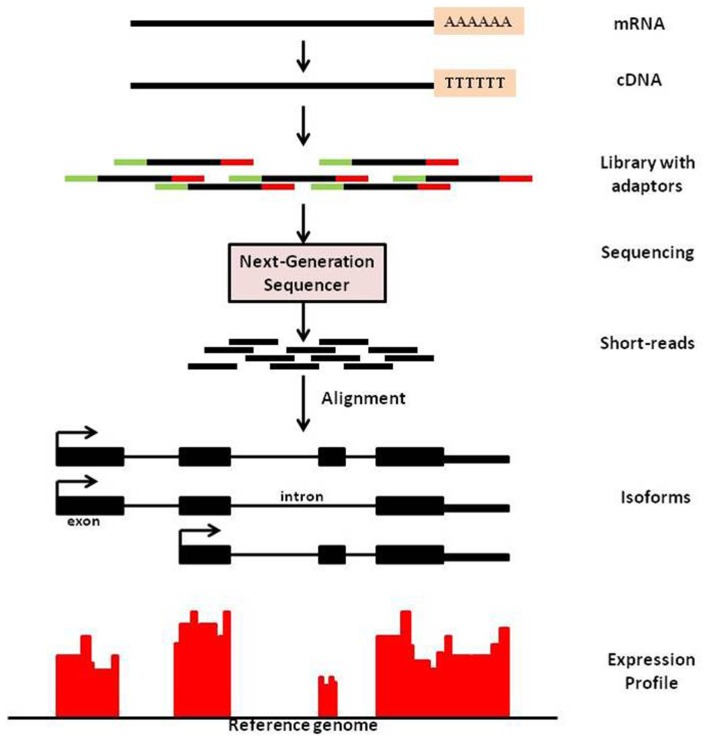
**An overview of mRNA-Seq experimental procedure**. For a typical mRNA-Seq experiment, mRNA is isolated and reverse-transcribed into cDNA libraries with homogeneous lengths (achieved by fragmentation). Adaptors at one or both ends of the RNA are added prior to cDNA amplification and library construction. New generation high-throughput sequencers enable to read from these fragments and these reads are then mapped to the genome of interest. Expression measure is then available as counts of reads mapped to that region.

Sequencing technology has advanced at a rapid rate in the past decade. The advent of massive parallel sequencing technologies, such as Illumina Genome Analyzer/Solexa, has revolutionized the genome-wide transcriptome studies leading to multiple applications. One such application is known as mRNA-Seq technology that provides a far more precise measurement of levels of transcripts and their isoforms than microarrays (Nagalakshmi et al., 2008). It is analogous to shotgun sequencing (Staden, 1979) used for whole genome, while it is being applied to transcripts and this method yields copy number of transcripts in a sample. In mRNA-Sequencing, RNA is isolated from sample; which is then reverse-transcribed to form cDNA, followed by fragmentation. New generation high-throughput sequencers enable to read from these fragments and these reads are then mapped to the genome of interest. Expression measure of any gene is thus available in digitized form through counts of reads mapped to that gene (See Figure 1 for an overview of mRNA-Seq experimental procedure).

With respect to reproducibility, this technique has been shown to be highly reproducible across technical replicates (Mortazavi et al., 2008). On the other hand if microarrays are used to measure expression of genes, it has been shown that the correlation between two replicates is in the range (0.5, 0.95; Draghici et al., 2006).

One of the main advantages of this data is in its quality. The eukaryotic genomes can be roughly thought to be constituted of RNA polymerase II transcribed and non-transcribed regions. In mRNA-Seq data the reads that map to the transcribed region (“exons” of a gene) represent the signal and background, whereas those coming from non-transcribed regions (“introns” or other inter-genic regions) can be assumed to be only background. Often the density of reads falling in the non-transcribed region would be of the order 10^−4^ whereas that for the coding region could be 10^−1^, which results in a signal to noise ratio of 10^3^.

Apart from being more reliable than the more popular microarrays, there are some very appealing advantages of this technique. For example, it actually produces sequence information of a transcriptome at a single nucleotide base-level. As a result we are able to determine splice junctions and alternative splicing events with high confidence. This technique has been used to investigate alternative splicing in several organisms, e.g., mammals (Mortazavi et al., 2008; Pan et al., 2008; Sultan et al., 2008; Wang et al., 2008), Yeast (Nagalakshmi et al., 2008), Plants (Lister et al., 2008; Filichkin et al., 2009).

### Studies on alternative splicing event

The recent interest in studying alternately spliced gene expression stems from the fact that more than 90% of human genes have been reported to be alternately spliced (Pan et al., 2008; Wang et al., 2008). Furthermore, variants of transcripts produced by same gene have been shown to be involved in wide range of pathways and could perform distinctly different functions (see Davuluri et al., 2008). These phenomena have been under study for decades; however recent advances in biotechnology have enabled us to study these at a genome level simultaneously. With recent techniques such as mRNA-Seq, high-density DNA microarrays, and existing methods like Sanger sequencing of ESTs and cDNAs, genome-wide studies on many species have been reported (high-throughput techniques: Lister et al., 2008; Mortazavi et al., 2008; Pan et al., 2008; Sultan et al., 2008; Wang et al., 2008; Filichkin et al., 2009; and Sanger sequencing: Zhu et al., 2003; Iida et al., 2004; Alexandrov et al., 2006; Campbell et al., 2006; Wang and Brendel, 2006; Chen et al., 2007; Ner-Gaon et al., 2007).

### Sources of variation

The statistical challenges in analyzing mRNA-Seq arise from many perspectives. While some sources of error are due to inherent problems with the technology, some are contributed at laboratory or experimental level while remaining are limitations of inference methods used.

As an example of technical limitations, mRNA-Seq data does suffer from non-uniformities, for example it is known to have biases toward certain base compositions (Dohm et al., 2008). Another such known limitation of this technique is that longer genes are more likely to be sequenced and also more likely to be declared differentially expressed (Oshlack and Wakefield, 2009). It has been demonstrated by the same authors that weighting the differential expression statistics by gene length can mitigate this effect.

There are known experimental errors as well during the creation of a sequencing library, seeding and preparation of the flow cell, and synthesis of the sequence reads (sequencing phase of the experiment). Depending on the data these errors may not pose any significant problem (see Bullard et al., 2010). They also observed that the well-known control lane used at the base-calling calibration procedure may not have any significant effect on differential expression call.

According to Bullard et al. (2010), the most prominent source of variation in differential call is the normalization or calibration method used on the mRNA-Seq data. In this paper we would carry out inference on isoform expression under different normalization techniques.

Calibration of data becomes essential to remove the effects of such courses of variations. In the following section we have provided detailed descriptions and discussions on the existing methods.

### Expression analysis

Often analysis stops at finding alternately spliced variants only and further research into quantifying their expression is not carried out. In this paper our primary objective would be use the mRNA-Seq data to infer expression at transcript, i.e., isoform level, while utilizing all existing information on splice variants.

In mRNA-Seq data, each transcript is covered by numerous sequence reads. Since these reads are generally short in nature (approximately 35 bases in the data set considered here) these typically do not reflect expression of a single transcript but is often shared by several transcripts. Majority of these would be splice variants; however there are other possibilities as well, which we will address later.

Reads not only from exon regions but also from splice junctions (span two exons) could be available, but they too suffer from similar limitation of being shared by multiple isoforms. In case a junction is unique to a transcript then the reads from splice junction may be used as indicative of the expression. Otherwise in a complex structure of multiple exon sharing between different transcripts, the reads mapping to splice junctions would have to be modeled additionally if they are to be used for inferring expression of the isoforms.

As has been pointed out by Zheng and Chen (2009) comparison of expression at the individual transcript isoform level requires jointly consideration of all sequence reads belonging to the same gene. Thus often it may not be possible to detect isoform level expression in any obvious method and requires statistical techniques that would help us obtain individual isoform expression from this mixture along with estimates of uncertainty.

In the following sections we present review of relevant literature on expression estimation, followed by the description of the proposed Bayesian latent variable model and its application to a real data set.

## Materials and Methods

mRNA-Seq technique provides read count for genomic segments, known as exons. These can then be utilized in various manner depending on the question of interest, more often than not, these are summarized at gene level. However we prefer to quantify them at the level of disjoint exons or parts thereof. The motivation is as followed by Jiang and Wong (2009), wherein isoforms for any gene share exons as a whole. Since in reality this is often not true we achieve this by slightly modifying the known exon constitution of that gene. This is achieved by considering disjoint parts of exons, which are shared and otherwise, individually as pseudo-exons.

### Data calibration

Since number of reads for any genomic region would naturally depend on the length of that region it is a common practice to normalize it using the length information. Quite often it is represented as reads per kilobase per million (RPKM) mapped reads (Mortazavi et al., 2008). Thus the two most influential factors behind any expression summary from the mRNA-Seq data is

1. *n_j_*: total number of mappable reads in the sample for the *j*-th region
2. *l_j_*: length of that region,

Then

$$RPKM_{i}=\frac{n_{i}}{\underset{j}{\sum }\ell_{j}n_{j}}\times \;10^{9}$$

Although the RPKM thus obtained can then be analyzed similar to methods used for microarrays, in-depth look into the sources of variations is required before inference.

Often in mRNA-Seq data, different lanes of a flowcell would represent different samples (could be biological replicates or could be distinct treatment samples). Each sample would produce different total read counts depending on sequencing depths. Thus, as described above, the common approach is to adjust individual expression counts for each sample by the total count for that sample: e.g., RPKM as described above or a hypergeometric model (Marioni et al., 2008). Bullard et al. (2010) have pointed out that, for the data considered by them, global normalization is heavily affected by a relatively small proportion of highly expressed genes. Since these are often not the same genes across samples this potentially could lead to biased estimates of differential expression over the different samples considered.

A common phenomenon for microarray data analysis lack of robustness of inference (on say differential expression call, or classification) due to change in analysis techniques in previous step of data analysis. Quite often a moderate change in algorithm for normalization can dramatically alter biological conclusions (Bhattacharjee et al., 2004). It has been shown by Bullard et al. (2010) that mRNA-Seq data is no exception from this problem and hence we feel needs extended study before robust conclusions of expressions can be made based on this data.

In literature, so far, the main methods proposed for calibration are

1. Global normalization,
2. Use of house-keeping genes, which is a familiar concept for micro array data,
3. Quantile normalization, which is also a well established method used for other high-throughput data (Irizarry et al., 2003).

It has been reported that the standard total count normalization results in low variation across samples and other technical sources of variation. However does not produce significant gain in terms of differential expression call, when compared with microarray data (Bullard et al., 2010).

The use of house-keeping genes has a sound biological reasoning. However in practice obtaining a reliable set which would provide consistent expression across samples and replicates might prove difficult. Regarding quantile-based normalization, Bullard et al. (2010) observe that for their data that this technique yields the most robust data without introducing additional noise. Thus for our purpose we would explore global and quantile-based normalization of our data.

### Modeling of expression in classical framework

The initial efforts of expression modeling have been to use the counts of reads mapped as the variable itself. This has lead to use of discrete distributions on most of the existing literature. However one should note that with complex normalization techniques being applied to mRNA-Seq data modeling the calibrated data with discrete distributions like Poisson or Negative Binomial may not be feasible. Nevertheless we provide a review of these models and corresponding inference procedures in the following.

#### Poisson modeling for expression analysis

Let *n_g_* be the count of reads mapped to gene *g*. Assuming that the reads are sampled independently and with replacement *n_g_* follows a Poisson distribution (Sultan et al., 2008), i.e.,

$$n_{g}\sim \;\text{Poisson}\left(\lambda_{g}\right),$$

where λ*_g_* is the mapping rate and therefore large λ*_g_* means high expression. This can be alternately arrived at by assuming a multinomial model for counts (*n*) of collection of all genes (or parts thereof). If *p_g_* is the probability of each read being mapped to a gene *g* then since *n* is large and *p_g_* is small, approximately *n_g_* ∼ Poisson(λ*_g_*) with λ*_g_* = *np_g_*. It has been shown that more than 95% counts can be modeled well by this Poisson model (Marioni et al., 2008). However, simple Poisson model cannot explain all the variance. One suggestion is to assign a Gamma prior to λ. This would lead to the Negative binomial model and there it might be harder to interpret the two parameters.

#### Poisson modeling for joint calibration and expression analysis

By introducing a normalizing factor *C_g_* in the Poisson model we can achieve calibration at global level. Then

$$n_{g}\sim \text{Poisson}\left(C_{g}\lambda_{g}^{*}\right),$$

where $\lambda_{g}^{*}$ : normalized expression level, *C_g_*: similar to the RPKM described earlier. For differential expression analysis we can treat *C_g_* as known, and interpret $\lambda_{g}^{*}$.

#### Generalized linear model for joint calibration and expression analysis

This model was recently proposed by Bullard et al. (2010). Therein a generalized linear model (GLM) framework for the expression modeling was carried out jointly in presence of parameters quantifying other systematic sources of variations, like total read count difference across samples. The Poisson-log GLM proposed by them is as follows:

$$\log\left(E\left[n_{ig}\;|\;d_{i}\right]\right)=\log d_{i}+\lambda_{a\left(i\right)g}+\theta_{ig};$$

where the natural logarithm of the expected value of the read count *n_ig_* for the *g*-th gene in the *i*-th sample is modeled as a linear function of the gene’s expression level λ*_a(*i*)_*g** for the biological condition *a*(*i*) as reflected in sample *i* plus an offset (log *d_i_*) and possibly other technical effects (þ*_ig_*). If no technical replicates are carried out, as is often the case, *a*(*i*) will be simply *i*.

#### Testing for differential expression

Under the Poisson model, often data is further transformed, e.g., Log, Arcsine (Mortazavi et al., 2008). This is followed by the testing methods developed for microarray methods, e.g., *t*-test, moderated *t*-test, to identify significant expression changes. Note that the arcsine-root transformation is suggested for variance stabilization of the per-gene read proportions within each sample (Marioni et al., 2008).

To test *H*_0_: λ*_g1_* = λ*_g2_* following are some examples of existing testing procedures that can be directly applied to this type of data. Following and extending notation introduced earlier,

1. Binomial Test (Ji et al., 2008)
  $$P\left(n_{g1}|\;n_{g1}+n_{g2}\right)\;\sim \;\text{Binomial }\left(n_{g1}+n_{g2}\;,\;\frac{C_{g1}}{C_{g1}+C_{g2}}\right)$$
2. Negative Binomial Test (Audic and Claverie, 1997)
  $$P\left(n_{g2}|\;n_{g1}\right)\;\sim \;\text{Negative Binomial }\left(n_{g1}+1\;,\;\frac{C_{g1}}{C_{g1}+C_{g2}}\right)$$
3. Chi-square goodness-of-fit test for Poisson counts (Mortazavi et al., 2008). Asymptotically,
  $$\underset{i=1,2}{\sum }\;\frac{{\left(n_{gi}-m_{gi}\right)}^{2}}{m_{gi}}\;\sim \;X_{1}^{2},\;$$
  where $m_{gi}=\frac{C_{gi}\left(n_{g1}+n_{g2}\right)}{C_{g1}+C_{g2}}$ are the expected observation under null.

For the GLM, three types of methods for differential expression inference have been proposed, viz. Fisher’s exact test statistic, likelihood ratio statistics based on a GLM, and *t*-statistics based on estimated parameters of the same GLM. All the methods have their merits and demerits, for example, distributions for the GLM-based statistics are derived under asymptotic theory; therefore, might be affected by small numbers of input samples or low counts (depending on which parameter is being tested). On the other hand although Fisher’s exact test does not make any assumptions on the sample size; it only adjusts for global experimental effects, unlike the likelihood ratio statistics adjust for general experimental effects as well as sample covariates. *t*-statistics on the other hand is severely affected by zero count in even one sample and is unable to detect differential expression in some of the obvious cases.

#### Adjusting for gene length bias in differential expression

It has been noted by several groups that longer genes contribute more to mapped sequence reads than shorter genes with similar expression. More importantly this may not be removed by scaling with length like RPKM (Mortazavi et al., 2008).

We investigated this feature for our data as following. We distributed the exons, irrespective of their gene, according to lengths into ten approximately equal size bins and calculated average RPKM for each of these bins. The pattern as reported by others seemed to be present for our data as well although not strongly (results not shown). There was a sharp drop at the last bin, which could be due to the fact that this bin contains exceptionally large exons, however there is always a physical limitation of number of reads coming from these exons, thus bringing down the RPKMs.

The effect of length bias has been noted on differential expression call also (Oshlack and Wakefield, 2009; Bullard et al., 2010). One possible explanation is the following. For a particular gene in two conditions let us increase the expressions from λ_1_, λ_2_ by *L* folds. Then the resulting *t*-statistics would be the following:

$$T=\frac{\left(\lambda_{1}L-\lambda_{2}L\;\right)}{\sqrt{\left(\lambda_{1}L+\lambda_{2}L\;\right)}}=\frac{\left(\lambda_{1}-\lambda_{2}\;\right)}{\sqrt{\left(\lambda_{1}+\lambda_{2}\;\right)}}\times \sqrt{L}$$

Thus if we measure more expression merely because of longer length we would also inflate the *t*-statistic artificially leading to possibly false differential expression call.

Some of the suggestions to overcome this are as follows. An *ad hoc* measure would be to consider read counts from a fixed length for each gene (Bullard et al., 2010). However this would results in loss of possibly huge part of the data and also difficult to ascertain what would be an appropriate length application to all genes and all scenario. Instead, as indicated above, re-weighting the *t*-statistic with length may be more effective. However, the problem of choosing a cut-off would need to be investigated.

### Bayesian modeling of mRNA-Seq data

In mRNA-Seq literature there has been some developments toward building Bayesian models particularly toward inferring isoform level expression. Zheng and Chen (2009) reported a fully Bayesian model whereas Jiang and Wong (2009) developed an empirical Bayes set up for isoform expression modeling.

Our proposed model will be closer to the model by Zheng and Chen (2009) in spirit. However there are significant differences in the two models and we will point these out as we describe our model. We analyze the mRNA-Seq data on *Arabidopsis thaliana* (Filichkin et al., 2009) using the proposed model.

The hierarchical Bayesian model developed by us can be used to infer about individual splice-variant (i.e., isoform) level expression of a gene and these can then be used to assess overall expression of a particular gene. The data used here for the purpose of illustration has been limited to uniquely mapped reads, however as has been mentioned earlier, by introducing an extended concept of (pseudo-)gene and (pseudo-)exon we can accommodate reads mapping to multiple locations as well. Thus this model can borrow information across different genomic locations belonging to same or different genes. For other methods to adjust for reads mapping to multiple genes see Li et al. (2010), Faulkner et al. (2008) and other relevant references therein.

The model presented here requires known structure of the splicing patterns of a gene. Although with some advanced modeling we are able to infer when the exiting knowledge of transcription variation is in question (thus leading to randomized-design matrix). Also by extending the design matrix would enable us to infer about yet unknown novel transcripts.

Additional advantage of the proposed method is that it is developed for multiple samples comparison unlike Zheng and Chen (2009) and also allows two-sample comparisons. Therefore we do not use latent variables for differential call instead we use latent variable as expression indicator. Thus for a particular gene and a specific isoform thereof we introduce at each sample level a latent variable that indicates whether the isoform is expressed in that particular sample or not.

This technique has twofold advantages over existing methods, at the level of model identifiability and also at the level of pairwise testing. Firstly, when genome level expressions of genes (and exons in this case) are explored the majority of them are not expected to express at every situation. Thus to represent the underlying biology realistically requires the distribution of the expression parameters to be able to take values very close to zero as well as large values. This often poses computational problems and one way to overcome this is to use the “spike-n-slab” type of model (Bhattacharjee and Sillanpää, 2005). By using a latent variable indicating whether an isoform is expressed or not produces the desired biological constraint on the model without introducing computational complexity. The advantage of these indicators for pairwise testing will be discussed later.

### Proposed model

For each exon *i* of a gene *g*, we model it’s expression (as measured by normalized reads there in) as a linear combination of expressions of the isoforms of that gene which share this particular exon. Thus,

$$y_{gil}=\underset{k}{\sum }I_{gkl}\beta_{gkl}X_{gik}+\varepsilon_{gil};$$

where *y_gil_*: normalized reads for the *i*-th exon in the *g*-th gene for the *l*-th sample, *I_gkl_*: latent indicator variable, which is one if the *k*-th isoform of the *g*-th gene is expressed in the *l*-th sample and it is zero otherwise, β*_gkl_*: latent variable measuring expression of the *k*-th isoform of the *g*-th gene is expressed in the *l*-th sample, *X_gik_*: the zero-one matrix indicating whether exon *i* is known to form part of the *k*-th isoform of the *g*-th gene, ε*_gil_*: residual error term for the *i*-th exon of the *g*-th gene in the *l*-th sample, *g*: ranges from 1 to *G*, the number of genes covered in the data (7737 genes from chromosome-1 of the *Arabidopsis* data), *i*: ranges over the number of exons for the *g*-th gene in the data, these exons could be biologically whole exons or disjoint parts thereof, (for the current data it ranges from 1 to 73), *k*: takes values 1 to the known number of isoforms for gene g (for our data values are between 1 and 10), *l*: represents different samples (for the data used for illustration there are five distinct treatment conditions with one sample each and a control sample to contrast with are available).

After careful exploration we chose multivariate Normal distribution for the errors; however for individual error a Gamma model might have been more effective in overcoming some biases in the data. Our choice of distribution was for the ease of computation.

In regard to the choice of error variance, our model assumes a sample × gene parameterization. Consider a gene with single isoform with *m* exons. Since the gene is known to have only one transcript thus if this transcript it expressed in a sample then all of its constituent exons will be expressed and should be expressed in similar amount. Thus any variation in the measurements *y_gil_* for *i* = 1, …, *m* would be purely noise in the measurement of expression of the gene at specific sample level. Thus our error modeling is clearly intuitive for single isoform genes which is majority of genes for this particular data (since 6510 genes of the 7737 genes have single isoform in this particular data). Now let’s consider the situation of a gene with multiple known isoforms. There, if a residual error is high that could not only indicate noise in measurement but also incomplete knowledge in all possible isoform formation of that gene, that is we could very well be missing a few isoforms and thus are unable to explain the observed expression measurement for some exons. Thus an assumption of gene *x* sample level variation would be realistic for such genes also.

This helps us in defining the likelihood of the data and to complete the hierarchical structure of the Bayesian model we restrict to known conjugate distributions as priors. In standard notation we define our model for the vector of expression of all exons pertaining to *g*-th gene for the *l*-th sample as: $Y_{gl}\;|\;I_{gl},\;\beta_{gl},\;\Sigma_{gl}\;\sim \;N_{m_{g}}\left(X_{g}\;\beta_{gl}^{*},\Sigma_{gl}\right),$ each element truncated below at zero; where, *m_g_* is the number of known exons for gene *g*; *X_g_*: is the known exon-isoform indicator matrix, dimension *m_g_* × *s_g_*; *s_g_* is the number of known isoforms for gene *g*;

$$\begin{array}{llll} \beta_{gl}^{*} & ={\left(\beta_{gkl}\;\times \;I_{gkl}\right)}_{s_{g}\times 1}; & & \\ \Sigma_{gl} & \equiv \text{diag }{\left(\frac{1}{\tau_{gl}},\;\cdots \;,\;\frac{1}{\tau_{gl}}\right)}_{m_{g}\times m_{g}}; & & \end{array}$$

τ*_gl_* ∼ Gamma(α_1_, α_2_) with mean α_1/_α_2_ and variance α_1_/(α_2_)^2^;

$\beta_{gkl}∼\text{Normal }\left(\mu_{gk}^{0},\frac{1}{\tau_{g^{k}}^{0}}\right)$, truncated below at zero and represents the expression of the *k*-th isoform of the *g*-th gene in the *l*-th sample;

$\tau_{gk}^{0}\;\sim \;\text{Gamma }\left(\alpha_{1}^{0}\;,\;\alpha_{2}^{0}\;\right)$ with mean $\frac{\alpha_{1}^{0}}{\alpha_{2}^{0}}$ and variance $\frac{\alpha_{1}^{0}}{{\left(\alpha_{2}^{0}\right)}^{2}};$

*I_gkl_* ∼ Bernoulli(*p*), is the latent indicators variable indicating whether the *k*-th isoform of the *g*-th gene is expressed in the *l*-th sample or not.

Using Markov-Chain-Monte Carlo samples for the parameters β*_gkl_*, *I_gk_*, τ*_gl_* to infer about these parameters and their functions of interest. Multiple choices of hyper-parameters $\left(\alpha_{1},\alpha_{2},\alpha_{{}_{1}}^{0},\alpha_{2}^{0},\mu_{gk}^{0},p\right)$ were carried out during the analysis of the data as a part of sensitivity analysis. In most cases reasonable choice of hyper-parameter values did not have major effect on the inference.

#### Computational aspects

We implemented our model in the WinBUGS software (Spiegelhalter et al., 1999) and used version 1.3. In our experience of this software, this particular version appears to perform better for large datasets with relatively lesser model complexity (when compared to other version). Typically two parallel MCMC chains were run starting from random initial values. Burn-in period for different models varied depending on which MCMC samplers were being used. Speed of convergence was not an issue for any of the models and attained target of 5% MCMC error with relatively small posterior sample (ranging in few hundred to few thousands depending on the parameter of interest). Apart from monitoring the MCMC error to assess convergence, the diagnostic tools provided within the WinBUGS software for checking convergence were used. Additionally, visual inspection of critical individual parameters is often found extremely useful to indicate convergence as well as to identify possible sources of un-identifiability in the model (or in the design matrix).

#### Inference

One of the main advantages of our model is that it has been developed for multiple conditions, however the parameterization enables us to infer at various level. With availability of data over a range of conditions differential expression call may be one of the many possible questions of interest regarding an isoform’s behavior. Thus instead of including a differential expression call indicator we model expression variability of an isoform across different conditions through the hyper-parameters ($\tau_{gk}^{0}$) in the hierarchical model. This setup allows us to identifying isoforms that are variable across different conditions. The profiles and clusters of isoforms can be studied using the β*_gkl_* parameters.

Moreover two-sample comparisons can be carried out with the help of Bayesian *t*-test. For Bayesian *t*-test, the posterior distribution of a *t*-statistic like random variable defined as following is derived for each pairwise comparison of interest. Differential call is made according to whether or not the 95% symmetric posterior probability interval (PPI) contains zero.

As has been reported by Bullard et al. (2010) classical *t*-test might suffer from inflated standard error estimate of a gene/isoform is not expressed in one of the two conditions. In our modeling framework we easily overcome this issue by utilizing the latent expression indicator variable. If the two latent variables indicate with high degree of confidence that an isoform is expressed in one condition and not in the other, then irrespective of expression amount it can be safely declared differentially expressed. This is a known knowledge in biology, that in order to play a significant role in a biological process a transcript may not have to be expressed in high degree but differently with high confidence.

Note that these latent indicator variables also serve the purpose of variable selection and regularization against over-fitting.

## Results

For illustration purposes we used the RNA-Seq data on *Arabidopsis thaliana* by Filichkin et al. (2009). High-throughput sequencing using the Illumina 1G platform (reviewed in Quail et al., 2008; Shendure and Ji, 2008) were carried out to capture transcriptomic expression information from a range of *Arabidopsis* samples. *Arabidopsis* tissues at different developmental stages and time points of the diurnal cycle were pooled to provide a broad view of expression within a specific condition. For five abiotic stress treatment conditions and additionally a control wild-type (WT) sample, RNA-Seq libraries were prepared and sequenced individually. The five conditions are Cold Stress (CS), Drought Stress (DS), heat stress (HS), High-light Stress (LS), and Salt Stress (SS).

The dataset was downloaded from NCBI GEO website (accession ID = SRP000935). Pooled data contains approximately 271 million. The gene information and other necessary resources of *Arabidopsis* genome were downloaded from TAIR9 database release available at http://www.arabidopsis.org. In house programs were made to extract isoform level information. Bowtie program was used to align the reads to genome and isoforms. We used 30-bases with up to 2-bases mismatch and unique match options to align the reads. The results presented in the subsequent sections will be based on data from *Arabidopsis* chromosome-1 only for limitation of space.

**Figure 2:**
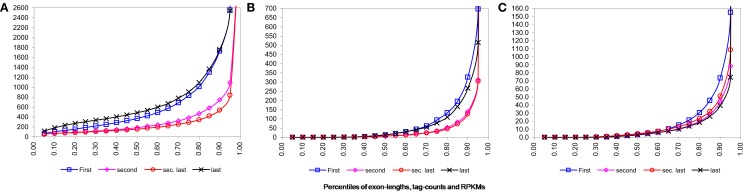
**Percentile distributions for first, second, second last, and last exons from transcription start site for single isoform genes**. Note that all the figures have been zoom-in for better visibility thus last percentiles have been omitted. **(A)** Percentiles of lengths of exons. **(B)** Percentiles of tag counts mapped to exons for wild-type plants. **(C)** Percentiles of RPKM, i.e., tag counts adjusted by length, for exons for wild-type plants.

For technologies like RNA-Seq, sequencing bias toward 3′ end is well-known. However for our data this effect is visible only moderately (see Figure 2 below). This could be due to the reason that we have utilized pooled data from two different primer techniques used in the original experiment. As reported by Filichkin et al. (2009) the 3′ bias is visible in the data where full-length (FL) enriched cDNA libraries were used, however coverage by the randomly primed library was more evenly distributed. Thus combining these two types of data probably have resulted in reduction of this effect on overall data.

**Figure 3:**
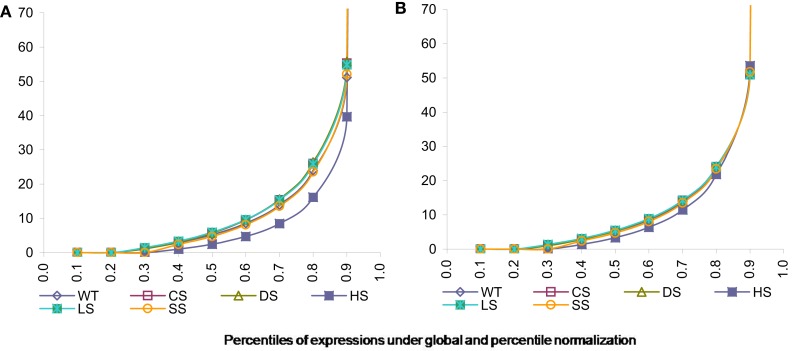
**Percentiles of expression data using global and percentile normalization for all six conditions, viz. WT, Wild-type-control; CS, Cold Stress; DS, Draught Stress; HS, Heat Stress; LS, High-light Stress; SS, Salt Stress**. **(A)** Percentiles of expression with global normalization. **(B)** Percentiles of expression with percentile normalization.

As can be seen from the following Figure 3A the data normalized using global calibration produces comparable quantiles for most conditions except for condition HS. We investigated the average expression within each percentile and observed that average expressions across all conditions (except HS) are comparable for all the percentiles using the global calibration. It appears under the HS condition there is a larger proportion of zero tag counts and also very high expression of a small proportion of exons. However in global calibration the total expression has been equated across all samples and thus it has forced all remaining percentiles for this condition (viz. HS) to be scaled down compared to others. We employ a percentile adjustment using the 10-th to 90-th percentiles of the globally normalized data and the resulting normalized data yielded comparable percentiles for all six conditions (Figure 3B).

However our quantile normalization would differ from that proposed by Bullard et al. (2010) in a number of ways. Firstly, we use ten percentiles and not quantiles. Secondly motivated by what we observe for the stress condition HS we believe that the total need not be scaled after doing the percentile normalization as has been suggested by them. It appears that for the HS condition up to the 90-th percentile data is comparable to other condition. So the exception in the top percentile is probably not an artifact but reflects the plant’s behavior under this stress condition. Thus we believe that it might be inappropriate to scale it down artificially.

Normalizing with house-keeping gene(s) could also be a possibility; however it requires relevant biological information which is not available in our case. Thus we proceed with comparing results based on two different types of normalization, global correction and percentile normalization.

**Figure 4:**
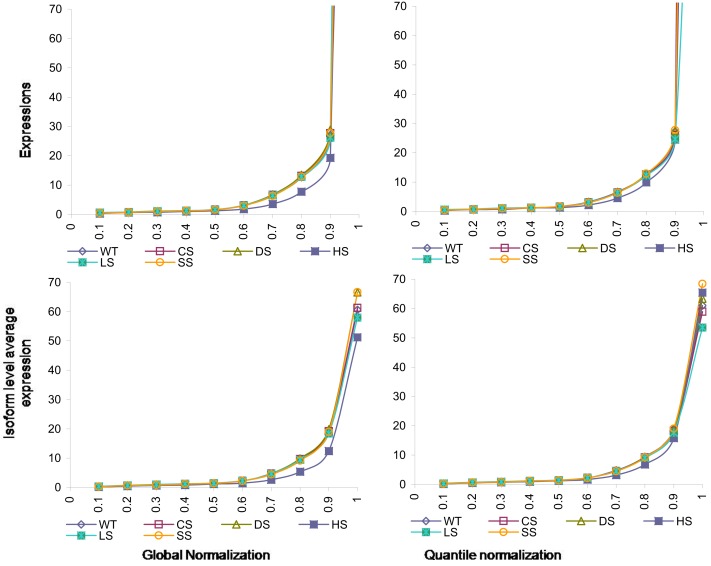
**Percentiles of isoform level expression using different normalization methods for all six conditions**. Top panel: expression percentiles under global normalization (left) and under quantile normalization (right). Bottom panel: isoform level average expression within each percentile under global normalization (left) and under quantile normalization (right).

The normalizations continue to have effects on isoform level expressions. In Figure 4 we present percentiles of isoform expressions, estimated by posterior means, under different conditions using the two normalization techniques. The last percentiles, which are large due to few handful isoforms with large expressions, are omitted from plots for better clarity. We also present the average expression within each of these percentiles for the different conditions and different normalization techniques.

Note that in our method of defining expression (at gene or isoform level) we utilize expression at exon-level. For this particular data, 74.2% of the genes (and 78.7% of isoforms) consisted of multiple exons, ranging from 2 to 73 exons for a single transcript. Amongst all isoforms only 12.9% consisted of 50% or more long exons, 28.5% have 50% or more short exons, and remaining had varied lengths of exons. We used 5-th and 95-th percentiles from the exon-length distribution to define short and long exons respectively. Thus in differential calling it is unlikely to have a systematic bias toward any length for these transcripts. However due to RPKM bias toward length the standard error will be affected by the underlying variation in length, weakening our power of detection. Another point to note is that for *Arabidopsis*, based on present knowledge, nearly 84% of the genes (on chromosome-1) are single isoform genes. However 69.4% of these genes have more than one exon.

Highly expressed isoforms and low/no-expression isoforms for each stress condition and for control sample were identified. A pairwise check for commonality of these when compared to other conditions were carried out (Table 1). We define low/no-expression if the estimated 95-th percentile of the Posterior Probability Interval (PPI) for an isoform expression is less than 10-th percentile of mean isoform expressions. Similarly an isoform is said to have high expression if 5-th percentile of its PPI is more than 90-th percentile of mean isoform expression. Thus with 95% posterior probability expression of an identified isoform will be within top or bottom 10% of isoform expressions. As has been noted earlier under HS the plants seemed to behave differently when compared with other stress conditions.

**Table 1:** **Pairwise comparison of condition-specific lists of isoforms with high and low expression, with high and low expression as defined in the text (normalization method used: global)**.

Pairwise count of common isoforms		Not expressed						
		WT	CS	DS	HS	LS	SS	Total
Highly expressed	WT		178	148	205	95	160	250
	CS	290		148	196	98	153	234
	DS	308	296		158	88	127	184
	HS	197	188	217		100	167	314
	LS	232	232	242	158		88	109
	SS	294	292	347	210	228		199
	Total	374	379	439	247	301	407	

*The entries above diagonal shaded in gray color are low-expressed genes and those below diagonal (not shaded) are highly expressed genes, in both conditions*.

We present differential expression behavior under different stress conditions when compared with expressions in the control sample (i.e., WT samples). A Bayesian *t*-like statistic was computed for each isoform in each such comparison. We consider a decision rule based on the mean of this statistic and compare it with an appropriate *t*-critical value based on the number of exons for that isoform. Note that this rule can only be used for isoforms with two or more exons.

Alternately we use the PPI of the *t*-statistics and define those as up or down regulated if the 95% PPI of the *t*-statistic is in the upper or lower 10 percentile of all (mean) *t*-statistics thus calculated (Table 2). This method then uses the uncertainty in estimating the *t*-values and would be applicable to all isoforms irrespective of number of constituent exons. The two method yields different but non-contradictory results (results not shown).

**Table 2:** **Up and down regulated isoforms for each stress conditions when compared with WT, using alternate definitions of differential expression**.

Stress condition ↓	Based on 95% PPI of *t*-statistics				Based on posterior mean of *t*-statistic and appropriate *t*-critical value			
Regulation →	Down		Up		Down		Up	
Normalization →	Global	Quantile	Global	Quantile	Global	Quantile	Global	Quantile
CS	100	120	91	71	39	54	54	42
DS	90	105	152	129	38	56	102	80
HS	467	319	55	94	571	364	27	61
LS	119	134	116	96	84	98	63	51
SS	78	84	156	149	73	73	51	51

It is apparent from the results presented so far that the isoform expressions vary under different stress conditions as expected. However many of the genes are not single isoform genes thus we need to explore further whether the observed variability is only at the gene level across conditions or whether alternately spliced transcripts behave differently under different conditions.

**Figure 5:**
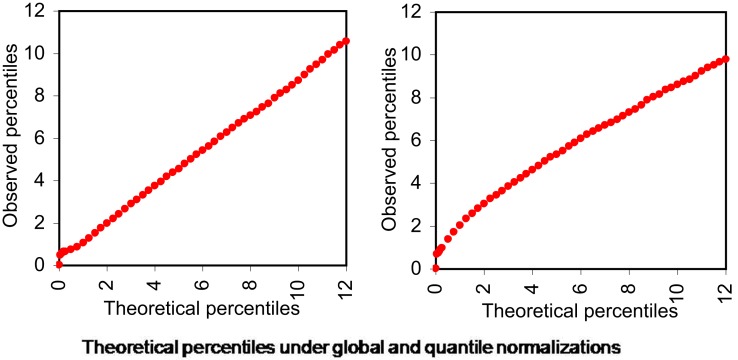
**Theoretical percentiles (in *X*-axes) and observed percentiles (in *Y*-axes) of overall isoform level expression variability parameters (across all six samples) under different normalizations**. Left panel: global normalization, right panel: quantile normalization.

Using overall variability parameters we estimate that approximately 4.06% of isoforms with globally normalized data and 2.74% isoforms with quantile normalized data exhibit varied expression under different stress conditions. To infer these we observe that the variability parameters estimated follow approximately Gamma distributions (Figure 5).

Of the total 7737 genes analyzed, 1227 genes have multiple isoforms. Of which 75 genes have at least one isoform not variable (i.e., overall variability parameter estimate is less than the 35-th percentile) and another moderately variable (i.e., variability is over the 65-th percentile). More interestingly 195 genes have at least one highly variable isoform (with variability above than the 90-th percentile) and another in the lower half. Thus for same gene not all isoforms behave in similar manner across all sample conditions. The above findings are based on globally normalized data and similar summary under quantile normalization are respectively 57 and 175 genes.

We compared our findings with two existing methods, namely modeling the exon-expression data with GLMs and secondly by using splice junction data only to infer isoform expression using Bayesian models.

**Figure 6:**
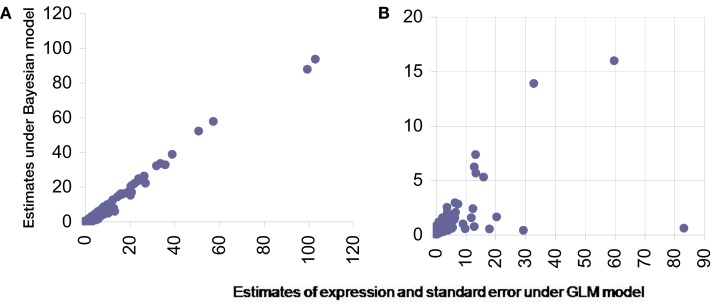
**Comparison of estimates from proposed Bayesian model (*Y*-axis) and GLM (*X*-axis)**. **(A)** Presents scatter plot of estimated expression levels for single transcript genes under the two methods while **(B)** presents the estimated standard error in these expression measurements from the same data under the same models.

For GLM we used a model similar to one proposed by Bullard et al. (2010). However due to the change in nature of data from discrete to continuous, induced due to normalization the error distribution for modeling in GLM was chosen to be Gaussian. The standard asymptotic theory based inference was carried out for this model. The mean estimates of the transcript level expression (for single transcript genes) were compared and were found comparable between the proposed Bayesian method and GLM (Figure 6A). To judge the quality of inference it is not just the point estimates that should be checked but also the confidence on these estimates. The estimated standard error around point estimates provides a well-known means of assessing the quality the estimated value. For this we firstly note that for single exon genes this can’t be estimated under existing procedures. However Bayesian paradigm does allow posterior inference with singleton data as well and hence an estimate for standard error for such genes too can be obtained. For other transcripts with multiple exons the Bayesian model consistently produces smaller standard error and thus outperforms the standard techniques like GLM (Figure 6B).

The Bayesian modeling framework proposed for exon data can be readily used for splice junction data too. Note that splice junctions would be typically short in length and as discussed earlier mRNA-Seq technology produces number of reads biased toward longer genomic regions. This is expected to cause the reads to have inflated amount zero read counts and underrepresentation the true expression from these regions. Our preliminary exploration of the splice junction data confirmed this hypothesis. We further compared the distributional behavior of the tag counts from the splice junctions to that based on reads from exons and found them to be comparable to those from exons of similar lengths.

**Figure 7:**
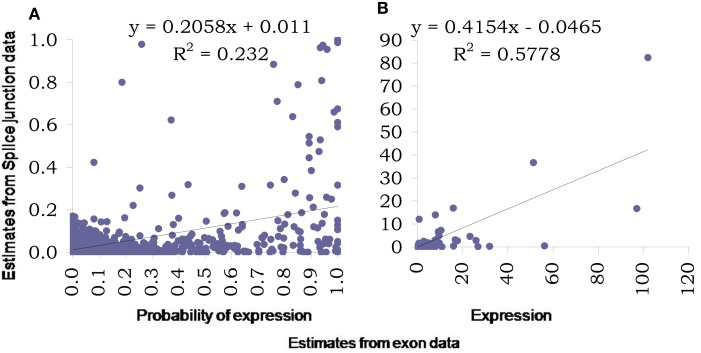
**Comparison of estimates using proposed Bayesian model from splice junction data (*Y*-axis) and exon data (*X*-axis)**. The left panel **(A)** presents scatter plot of estimated probabilities of isoform expression based on the two data types while the right panel **(B)** presents the estimated expressions at isoform level from the two data types. Both graphs also include fitted regression lines, corresponding equations and *R*^2^ values.

For further analysis of the splice junction data, it was first normalized using a similar percentile normalization technique described earlier and then modeled using the proposed Bayesian modeling framework. Figure 7 presents comparison of estimates obtained using this model based on data from exons with those from based on data from splice junctions. The two key outcome of the proposed model is assessing the probability of a transcript being expressed and the corresponding expression (posterior estimates in Figures 7A,B respectively). In addition to plotting the pairs of estimates, regression lines were fitted and corresponding equations (along with *R*^2^) were presented in the figures to provide an assessment of linear association between the two sets of estimates.

As was noted from the raw data itself, the splice junction data underestimates transcript presence and thus the estimated probabilities based on junction data are much lower than those obtained using exon data. This relation is curvilinear in nature and possibly is “S-shaped” as has been observed elsewhere, e.g., for microarrays. The *R*^2^ measure reflects this low degree of linearity between the two estimates. The expression estimates are affected also but the degree of linearity is much improved (with a reasonably high *R*^2^), although still continues to under-estimate. We also explored the inherent variability in splice junction data and found it to be higher compared to exon-based-data. This can be assessed easily by comparing measurements on exons and junctions from genes with single transcript only (results not shown).

## Discussion

We have presented a Bayesian framework based on mRNA-Seq data to infer expression at isoform level. One of the main advantages of our model is that it has been developed for multiple conditions, however the parameterization enables us to infer at various levels. Although complex experimental scenarios are not uncommon, as can be seen from the real data sued here for illustration, a lot of the focus on methodological development for mRNA-Seq data continues to focus on differential expression (e.g., Wang et al., 2010; Kadota et al., 2012, etc). The exclusion of differentially expressed genes for better calibration is similar to exclusion of the extreme tails in percentile based normalization method used here. The main difference being in presence of more than two condition differential expression may not be a useful criteria however percentiles would still provide a measure of extreme to be excluded from calibration.

The modeling setup allows inference at gene level (in terms of expression or its variability), at transcript level, comparison across samples, comparison between samples, etc.

We have utilized latent indicator variable for expression apart from additional parameters to quantify such expression. This differs significantly from existing approaches, where latent variables are used for differential call. Our method has twofold advantages over existing methods. First advantage is at the level of model identifiability, which is well-known for this type of spike-n-slab models. These variables also serve the purpose of variable selection and regularization against over-fitting.

Secondly, our method has an advantage at the level of pairwise testing. If an isoform is not expressed in one of the samples then this would typically un-stabilize a formal *t*-statistic, however can be captured here using the latent expression indicators. This is a known in biology, that in order to play a significant role in a biological process a transcript may not have to be expressed in high degree but differently with high confidence. Thus if for an isoform the latent expression variables indicate with high confidence expression in one condition and not expressed in another the corresponding isoform can be declared differential, without the help of a *t*-statistics.

We would like to reiterate that focus of this paper has been in developing modeling and inference procedure for expression measurement. Thus several other relevant aspect of mRNA-Seq data has not been addressed. Some of these are, how to use splice junction reads, what to do with multi-reads, how to make better use of unmapped reads. From current literature we see that a considerable effort is being put on these issues [Top-Hat and Cufflink by Trapnell et al. (2012), FX by Hong et al. (2012)], however comparatively less on what to do once we have satisfactorily mapped the reads and have been able to derive the alternate splicing structure.

Our aim has been to make the readers aware that, firstly there should be a modeling setup that allows complex experimental data going beyond differential expression to be analyzed, secondly even for simplest of inference, like expression of a single transcript gene; there could be significant effect of normalization procedure. We believe the proposed model and inference setup here addresses these adequately.

For the particular data used for illustration here, we noted that there could be significant effect of normalization, if not for all, at least in part of the data. For this data an in-depth presentation of the differential and variable expression could not be provided here. However it was known that alternate splicing plays a very important role in functioning of *Arabidopsis* and for many genes multiple splice variants were found to be active under different conditions.

## Conflict of Interest Statement

The authors declare that the research was conducted in the absence of any commercial or financial relationships that could be construed as a potential conflict of interest.
